# An integrative investigation of sensory organ development and orientation behavior throughout the larval phase of a coral reef fish

**DOI:** 10.1038/s41598-021-91640-2

**Published:** 2021-06-11

**Authors:** John E. Majoris, Matthew A. Foretich, Yinan Hu, Katie R. Nickles, Camilla L. Di Persia, Romain Chaput, E. Schlatter, Jacqueline F. Webb, Claire B. Paris, Peter M. Buston

**Affiliations:** 1 Department of Biology and Marine Program, Boston University, Boston, MA 02215 USA; 2 Department of Ocean Sciences, University of Miami’s Rosenstiel School for Marine and Atmospheric Science, Miami, FL 33149 USA; 3 Department of Biological Sciences, University of Rhode Island, Kingston, RI 02881 USA; 4 Department of Biology, Colorado State University, Fort Collins, CO 80523 USA; 5 Division of Biological Sciences and Engineering, Red Sea Research Center, King Abdullah University of Science and Technology, Thuwal, 23955-6900 Kingdom of Saudi Arabia; 6 Department of Biology, Boston College, Chestnut Hill, MA 02467 USA; 7 Present Address: School of Biological Sciences, Victoria University of Wellington, Wellington, 6140 New Zealand

**Keywords:** Animal migration, Behavioural ecology, Ecology, Marine biology, Animal migration, Behavioural ecology, Population dynamics

## Abstract

The dispersal of marine larvae determines the level of connectivity among populations, influences population dynamics, and affects evolutionary processes. Patterns of dispersal are influenced by both ocean currents and larval behavior, yet the role of behavior remains poorly understood. Here we report the first integrated study of the ontogeny of multiple sensory systems and orientation behavior throughout the larval phase of a coral reef fish—the neon goby, *Elacatinus lori.* We document the developmental morphology of all major sensory organs (lateral line, visual, auditory, olfactory, gustatory) together with the development of larval swimming and orientation behaviors observed in a circular arena set adrift at sea. We show that all sensory organs are present at hatch and increase in size (or number) and complexity throughout the larval phase. Further, we demonstrate that most larvae can orient as early as 2 days post-hatch, and they swim faster and straighter as they develop. We conclude that sensory organs and swimming abilities are sufficiently developed to allow *E. lori* larvae to orient soon after hatch, suggesting that early orientation behavior may be common among coral reef fishes. Finally, we provide a framework for testing alternative hypotheses for the orientation strategies used by fish larvae, laying a foundation for a deeper understanding of the role of behavior in shaping dispersal patterns in the sea.

## Introduction

Natal dispersal, the movement of offspring away from their parents and subsequent settlement in a new habitat, has the potential to connect distinct and often distant populations with important consequences for population dynamics and gene flow^1^. Most coral reef invertebrates and fishes produce dispersive larvae that leave the reef and develop in the upper water column for several days to months before locating and settling in appropriate benthic habitats. The distance over which these larvae disperse is ultimately determined by the interaction of ocean currents and larval behavior^2^. Due to their diminutive size, marine larvae were once thought to disperse passively by drifting with ocean currents to reach settlement habitats that may be far from their natal origin^3^. However, there is empirical evidence that the late-stage larvae of many marine fishes have strong swimming abilities^4–8^ and can orient their movements^9–13^ in response to one or more types of sensory cues^14–20^. This suggests that the behavior of late-stage larvae plays an active role in determining their ultimate dispersal trajectories^21^. However, such behaviors may not be limited to late-stage larvae. Biophysical models predict that if larvae can orient their swimming behavior soon after hatching, then they will experience higher rates of survival to settlement compared to larvae that do not begin to orient until later in the larval phase and will be favored by natural selection^22^.

The age at which orientation behavior begins is assumed to be limited by the functional capabilities of their sensory systems as well as their swimming abilities, which allow larvae to detect environmental cues and orient their movements, respectively. It has often been stated that larval fishes have “well-developed” sensory systems (e.g., Ref.^23,24^) or that sensory acuity increases during ontogeny (e.g., Ref.^25^). However, the anatomy of one or multiple sensory systems has been described in very few species from hatch through the entire larval phase (reviewed in [olfaction and taste]^26^; [lateral line]^27^). Furthermore, only a few studies have attempted to integrate developmental sensory anatomy with the experimental analysis of the ontogeny of behavior in any one species (e.g., Ref.^28–30^). In addition, to our knowledge, no empirical studies have simultaneously evaluated the ontogeny of multiple sensory systems, swimming abilities, and orientation behaviors throughout the larval phase due to the challenges faced in collecting or rearing an ontogenetic series of any reef fish species. Here we address this knowledge gap, conducting the first integrative study of the development of multiple sensory systems and the ontogeny of behavior, from hatch to settlement, during the larval phase of a coral reef fish.

We used the neon goby *Elacatinus lori* as a study system. *Elacatinus lori* is a member of the most speciose family of marine fishes (Gobiidae) and is a cryptobenthic reef fish endemic to the Mesoamerican Barrier Reef^31–34^. Adult *E. lori* lay demersal eggs on the inner wall of tube sponges^35^. After hatching, the larvae leave the sponge and develop in the upper water column for 26 ± 3.6 d before locating reef habitat and settling on a tube sponge^36–38^. The estimated median dispersal distance for *E. lori* larvae is 1.7 km with no observed dispersal events exceeding 16.4 km^36^, and these estimates of dispersal have been validated via sibship reconstruction^39^. The extent of larval dispersal in *E. lori* ^36^, which is relatively restricted compared to other species^40–42^, combined with the observation that their swimming abilities improve throughout development^8^, suggests that the behavior of *E. lori* larvae may actively influence their dispersal trajectories.

To investigate the ontogeny of larval sensory systems, swimming abilities and orientation behaviors, we used SCUBA to collect *E. lori* embryos from tube sponges located on a transect offshore from South Water Caye, Belize. Along this transect, yellow tube sponges *Aplysina fistularis*, each containing a single breeding *E. lori* male, were surveyed daily to determine the presence/absence and stage of development of *E. lori* embryos within each sponge. Eggs were collected from a sponge on the day prior to natural hatching and the fish hatched immediately upon collection. Newly-hatched, wild-caught larvae were reared from hatch through settlement in a field-based lab^35^ on South Water Caye, Belize to determine the timing of the ontogeny of: (1) all major sensory organs (visual, lateral line, auditory, olfactory, and gustatory), (2) swimming abilities (critical swimming speed [U_crit_] and turning angle), and (3) orientation behaviors.

## Results and discussion

### Multiple sensory systems increase in complexity throughout the larval phase

**Figure 1:**
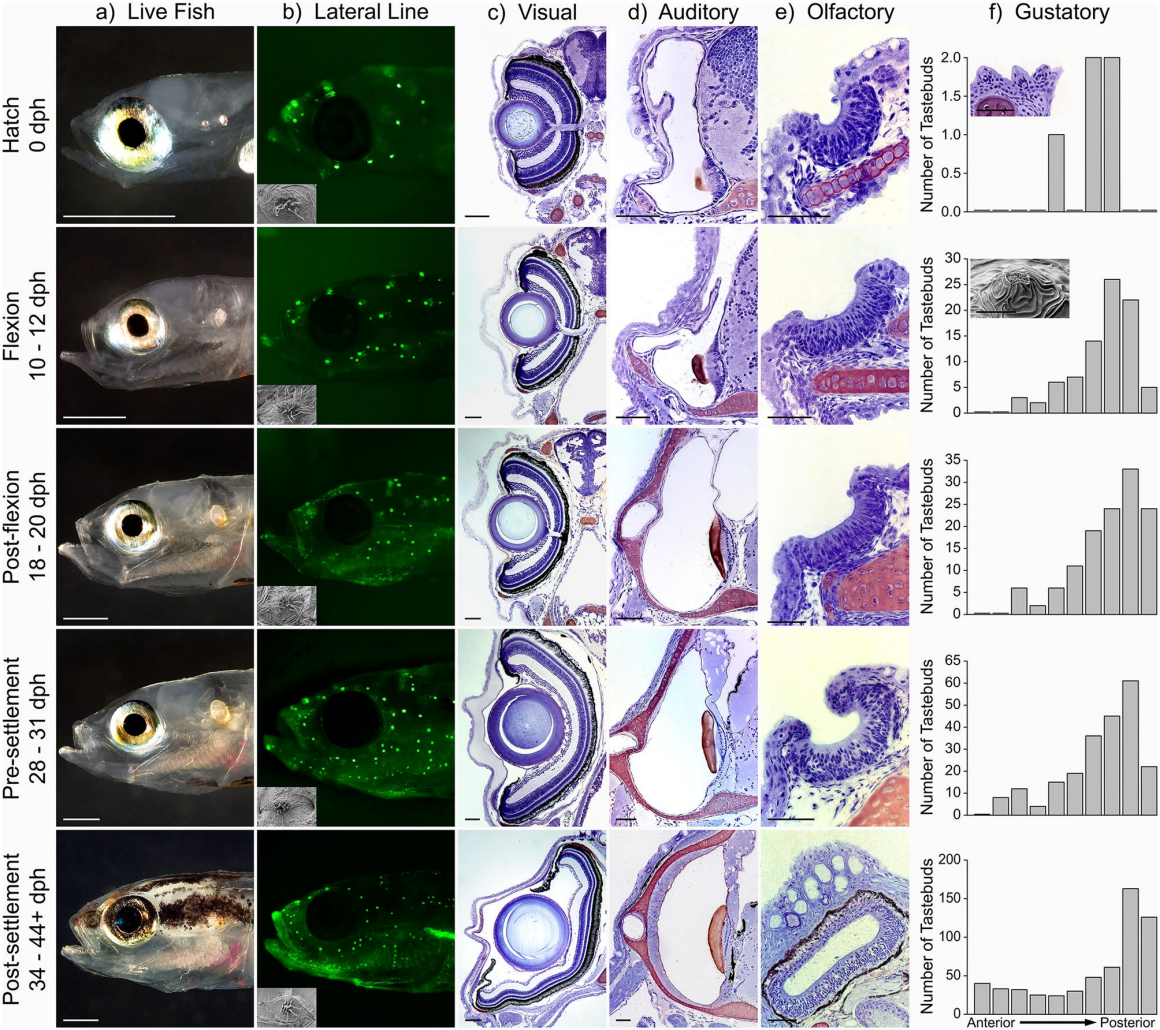
Development of the lateral line, visual, auditory, olfactory and gustatory sensory organs in *E. lori* larvae (day of hatch [0 days post-hatch (dph), 3–3.4 mm notochord length], flexion [10–12 dph, 4–5 mm standard length (SL)], post-flexion [18–20 dph, 5–7 mm SL], pre-settlement [28–31 dph, 7–9 mm SL]) and post-settlement juveniles (34–44+ dph, 9–15 mm SL) using live imaging (**a**), vital fluorescent imaging (**b**) scanning electron microscopy (**b**,**f** insets), histology [(**c**)–(**e**); transverse sections of whole fish; (**f**) inset] and data derived from histology (**f**). (**a**) Live larvae (0, 10, 20, 28 dph) and a post-settlement juvenile (38 dph). Note: change in the relative size of the eye, and onset of pigmentation in 34–44+ dph fish. Scale bars: 500 µm. (**b**) Neuromast receptor organs of the lateral line system in larvae, which increase in number, and are in well-defined lines in older larvae, as well as in post-settlement juveniles, in which a small number of neuromasts above the eye and on the cheek will be enclosed in pored lateral line canals. Insets illustrate the morphology of neuromasts, which take on a diamond-shape; hair cells (ciliated) located in the central region of the neuromast. The elongated gelatinous cupula in which cilia of hair cells are embedded was removed during preparation to visualize the hair cells. (**c**) A prominent, well-developed eye is present at hatch and is already comprised of a multilayered retina (layers increase in thickness at different rates through the larval phase), a spherical lens, and a cornea (0–30 dph); the optic nerve is visible in images of 0–20 dph fish, but is out of the plane of section in the 28–44+ dph fish. Note: corneal thickness in the 28–31 dph fish is a preparation artifact. Scale bars = 50 µm. (**d**) Inner ear is present at hatch (0 dph). The sensory epithelium of the sacculus (the largest of the three otolithic organs, the other two not visible in plane of section) is located on the vertical wall (lateral to the hindbrain) at all stages; it is overlaid by a calcareous otolith (otolith remnant is dark pink). The horizontal semicircular canal (one of the three semicircular canals) is well-formed by 18–20 dph; it is visible in cross section at the lateral edge of the cartilaginous neurocranium (dark pink). Scale bars = 50 µm. Note: otoliths (especially the large saccular otolith) are visible in live larvae [(**a**) 0–31 dph] but are obscured by pigment in post-settlement juveniles (34–44+ dph). (**e**) The olfactory organ is a patch of ciliated olfactory neurons (cilia not visible at this resolution) on the surface of the “snout” at hatch; it gradually invaginates and increases in length and width during the larval stage, and is enclosed in a blind sac with two nares (not in plane of section) in post-settlement juveniles. (**f**) Internal taste buds are present at hatch in the posterior half of the oral cavity (including the gill arches); their number increases (note Y-axis values) and their distribution expands anteriorly to occupy all internal surfaces of the oral cavity and the surface of the lips. Insets: top—three taste buds on gill arch (Scale bar = 50 µm); bottom—one taste bud (just behind the teeth of the lower jaw) showing microvilli at the tips of the sensory cells that comprise the taste bud, which is surrounded by general epithelium (Scale bar = 5 µm). Some images have been transposed (left to right), or partially rotated for uniformity. See text and SI Table 1 for additional details.

Morphological analyses of each of the major sensory organs (lateral line, visual, auditory, olfactory, and gustatory) were carried out using lab-reared fishes (Fig. 1a). Histology, vital fluorescent staining (4-di-2-ASP, a mitochondrial stain that labels neuromast receptor organs of the lateral line system^43^), and scanning electron microscopy (SEM) were used to assess the morphology and/or number of sensory organs (Fig. 1b–f). Analysis of the morphology of the sensory organs in *E. lori* indicated that the ontogenetic trajectories of each of the sensory systems are unremarkable in comparison to those in other fishes (with some minor exceptions), but some functional inferences could be made.

At hatch, the mechanosensory lateral line system is comprised of 22 diamond-shaped neuromast receptor organs located in the epithelium of the head and trunk (Fig. 1b: 0 dph), and neuromast number increases gradually throughout the larval phase. Unlike the round neuromasts found in the larvae of other species (e.g., zebrafish^44^; cichlids^45^; reviewed in^27^), *E. lori* larvae have distinctive diamond-shaped neuromasts (Fig. 1b, insets) with overlying cupulae that have “wing-like” projections that reach the tips of the neuromast (see Fig. 6, panel C in reference^27^); these are predicted to enhance sensitivity to water flows. In late-stage larvae, a small number of neuromasts become enclosed in only a small number of short, pored, ossified lateral line canals on the head (a reduced canal phenotype)^25^. Other, more numerous neuromasts are located on the skin, some of which are the homologues of canal neuromasts present in ancestral species that have a more complete set of well-ossified canals, like many other fishes^46^. A rapid proliferation of neuromasts on the skin during the larval stage in *E. lori* likely enhances overall sensitivity to the velocity component of water flows^27^. At settlement, several hundred neuromasts are found in well-defined lines on the skin of the head, trunk and tail (Fig. 1b: 28–31 dph)^27^, a complex pattern that is typical among gobies^47,48^ but uncommon among other fishes.

At hatch, a prominent eye is comprised of a spherical lens, a thin cornea, and a pigmented, multilayered retina, indicating that it is functional. The different retinal layers increase in thickness at different rates during the larval phase (Fig. 1c)^49^ but the ratio of lens to eye diameter remains constant (0.40 ± 0.02 SD; as in the black bream, *Acanthopagrus butcheri*^50^). The lens is in contact with the retina at hatch but is separated from the retina by 20 dph (Fig. 1c: 18–20 dph). The position of the lens relative to the retina is correlated with the development of the accommodatory retractor lentis muscle in *A. butcheri*^50^. The ability of this muscle to control lens position allows the eye to focus as the focal ratio (focal length:lens radius) decreases^50^. This suggests that capabilities for image formation likely change through the larval stage^49^.

At hatch, the inner ear is comprised of several sensory epithelia composed of sensory hair cells—located within each of the three semicircular canals (the cristae) and within two otolithic organs (utriculus, sacculus). A calcareous otolith sits on top of the sensory hair cell populations in a small horizontal utriculus and a larger vertical sacculus (Fig. 1d: 0 dph). The third, and smallest of the three otolithic organs, the vertically-oriented lagena appears just caudal to the sacculus by 10 days post-hatch (Fig. 1a: 10–12 dph), the approximate time of caudal fin flexion^35^. The sensory epithelia in the three otolithic organs increase in size. In the sacculus, the sensory epithelium elongates in the rostro-caudal axis through the larval stage (Fig. 1d), presumably with an increase in the number of sensory hair cells^51–54^.

At hatch, the olfactory system of *E. lori* consists of two flat patches of ciliated olfactory neurons on the snout, which increase in size but do not develop lamellae, and near settlement, gradually become enclosed in blind sacs (the olfactory organs), each bearing an incurrent and excurrent naris (Fig. 1e)^26^. The timing of this transformation predicts when active ventilation of the olfactory epithelium (“sniffing”, or cyclic passage of water over the sensory epithelium) may start^51^. Thus, it appears that only the settlement stage larvae and newly-transformed juveniles are able to use their olfactory system to actively sample their chemical environment.

At hatch, a small number of taste buds are already found within the oral cavity and their number increases throughout the larval phase, appearing first on the gill arches, and then gradually on all internal surfaces within the buccal cavity and on the “lips” (Fig. 1f)^26^; this pattern was also noted in two damselfishes and a cardinalfish (Webb unpubl. data) and in some non-coral reef taxa^55,56^. It has been suggested that prior to the onset of a mechanism for active olfactory ventilation, normal cyclic respiratory ventilation, which brings a consistent flow of water through the buccal cavity and across the gills, may also bring water into contact with taste buds^26^. Thus, while olfaction is generally considered to be the chemosensory mode that likely initiates larval orientation behavior in the open ocean^16^, the gustatory system may provide a reliable chemical sampling mechanism that could also initiate orientation towards a reef^26^.

In sum, we found that the end organs of the lateral line, visual, auditory, olfactory, and gustatory systems are all present at hatch and increase in complexity during the larval phase, with respect to the size, number and/or distribution of sensory receptors. The overall ontogenetic trajectory followed by each sensory system is typical of teleost fishes, whether found on coral reefs or in other marine and freshwater habitats, as revealed by the few studies that have detailed the ontogeny of those individual sensory systems (e.g., Ref.^23,28,57,58^). However, it should be noted that some features of the sensory systems in *E. lori* are typical of gobies, but are relatively unusual among other fishes. These include reduced (developmentally truncated) lateral line canals accompanied by the proliferation of superficial neuromasts, absence of complex folding (lamellae) of the olfactory epithelium, and presence of a large sacculus containing a particularly large square saccular otolith in the inner ear^26,27,47,48^. Further analysis of sensory anatomy can be found in recent papers on the ontogeny of the olfactory organs and taste buds^26^, and mechanosensory lateral line^27^.

When comparing *E. lori* to other coral reef fishes, the morphology of the sensory organs suggests that the sensitivity of the lateral line system to water flows and ear to acoustic stimuli are enhanced (due to proliferation of superficial neuromasts and presence of a large saccular otolith, respectively), but the sensitivity of the olfactory system to chemical cues may be limited due to the absence of an active ventilatory mechanism. This may help to explain why settlement stage larvae use visual, but not chemical cues, to locate their preferred tube sponge hosts at settlement^37^; this is in contrast to other reef fishes that use olfactory cues to locate settlement habitat^59,60^. It should be noted that, like the pelagic larvae of most marine fishes, the larvae of *E. lori* have an inflated swim bladder, but like most other gobies, the swim bladder is lost after settlement. Thus, *E. lori* larvae should be able to detect sound pressure in the far field (at a distance from the reef) transduced by the swim bladder, but will lose this ability after settlement leaving them sensitive only to near field acoustic stimuli that directly stimulate the ear. Finally, it is likely that multimodal integration of sensory inputs is critical for the formulation of behaviors and that the relative contribution of the different sensory systems and the different types of cues to which they respond, changes through time as larvae orient toward and approach potential settlement habitat.

### Larvae swim faster and straighter throughout the larval phase

**Figure 2:**
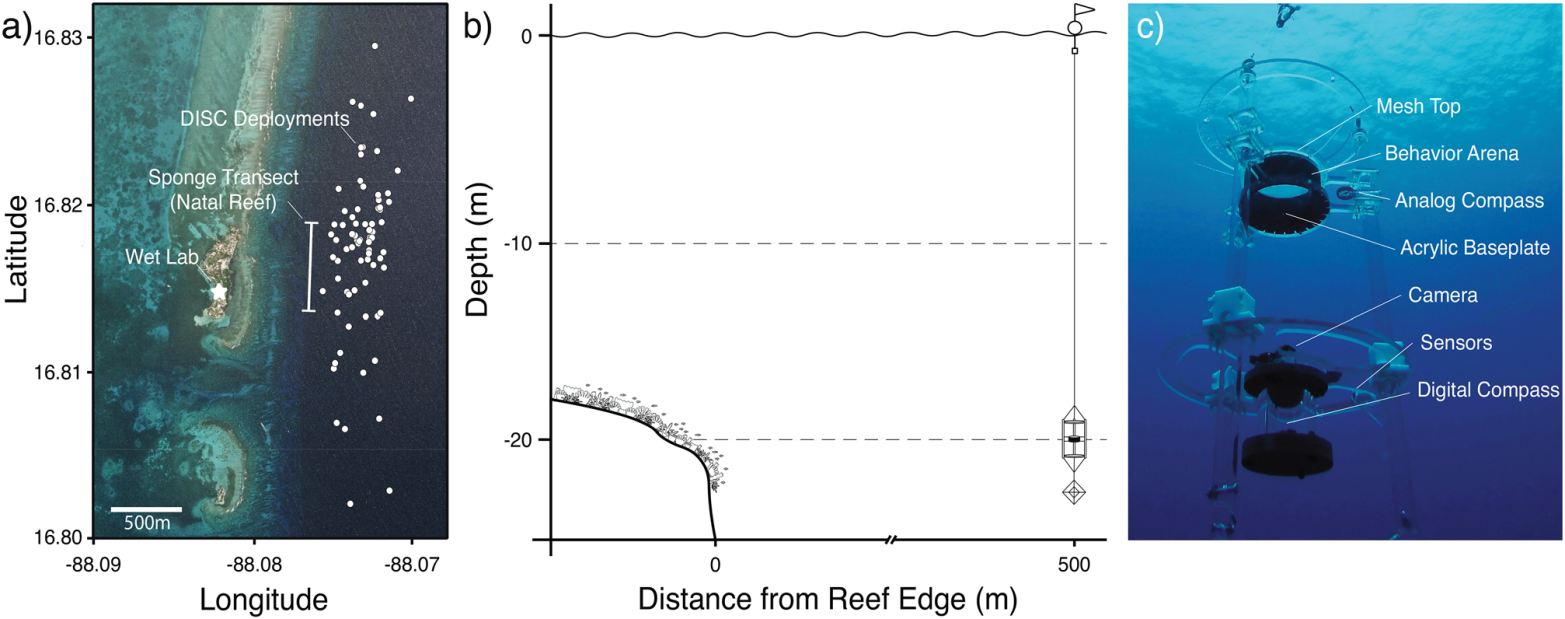
Methods for testing the swimming and orientation behavior of *Elacatinus lori* larvae. (**a**) Position of Drifting In Situ Chamber (DISC) deployments offshore from Southwater Caye, Belize. Satellite imagery was obtained from an ArcGIS Online basemap^87^. (**b**) A single larva was deployed in the DISC at 19 m depth in open water within 500 m of their natal reef. (**c**) Image of the DISC configured for assessing the behavior of *E. lori* larvae. Star—International Zoological Expeditions field lab. Bar—sponge transect where larvae were collected (i.e., their natal reef). White Dots—starting location of deployments (*n* = 120).

To investigate the ontogeny of swimming and orientation behavior in situ, wild caught *E. lori* larvae that were reared in a field-based lab were deployed just offshore from the Belizean Barrier Reef (Fig. 2a,b) in a Drifting in situ Chamber (DISC^12^, Fig. 2c), which was designed to observe and quantify larval fish orientation behavior in response to cues in the epipelagic zone^10,12,16,18,61–63^. Individual larvae, 2–30 days post-hatch (dph), were deployed approximately 500 m offshore from their natal reef (i.e., the sponge transect where eggs were collected, Fig. 2a,b) over a period of 4 months (120 successful deployments; SI Table 1). The position of a larva within the DISC was recorded continuously using video and individual frames were subsampled at 1 s intervals for analysis. The instantaneous swimming speed of a larva was calculated by measuring the distance it moved between sequential frames. Turning angle was calculated as the angle produced by three consecutive points on its trajectory.

**Figure 3:**
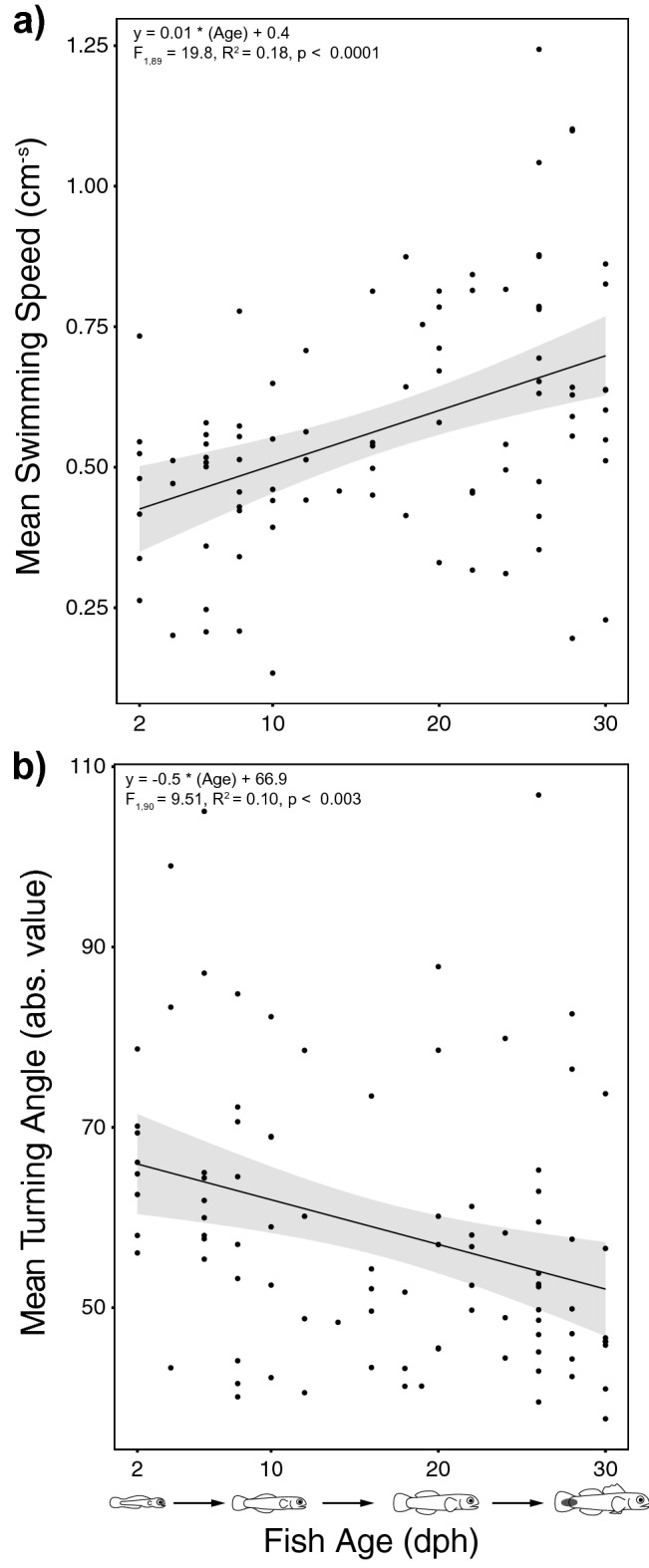
Ontogeny of swimming abilities. *Lines* represent the relationship of fish age to (**a**) mean swimming speed, and (**b**) mean turning angle measured from *E. lori* larvae deployed in the DISC. Shaded regions represent the 95% confidence intervals.

Mean swimming speed (Fig. 3a) increased and mean turning angle (Fig. 3b) decreased through the larval phase. The mean instantaneous swimming speed measured in the DISC (e.g., 2 dph: 0.45 cm s^−1^; 30 dph: 0.62 cm s^−1^) was less than the mean critical swimming speed (U_crit_) of similarly aged, lab-reared larvae tested in a swimming flume (e.g., 0 dph: 2.7 cm s^−1^; 30 dph: 7.2 cm s^−1^)^8^. Thus, laboratory-based swimming flumes provide a forced (i.e., rheotaxis induced) metric of swimming performance, likely overestimating a larva’s routine swimming speed. In contrast, the DISC provides an unforced (i.e., not induced by rheotaxis) metric of in situ swimming behavior, likely underestimating a larva’s routine swimming speed. However, taken together, DISC and flume data suggest that *E. lori* larvae are capable of swimming at speeds comparable to mean current speeds in the study area (4.38 ± 2.72 cm s^−1^ [mean ± SD]; calculated from the drifting speed of the DISC during deployments at 19 m depth)^64^ that would allow them to influence their dispersal trajectory.

### Orientation behavior begins shortly after hatching

**Figure 4:**
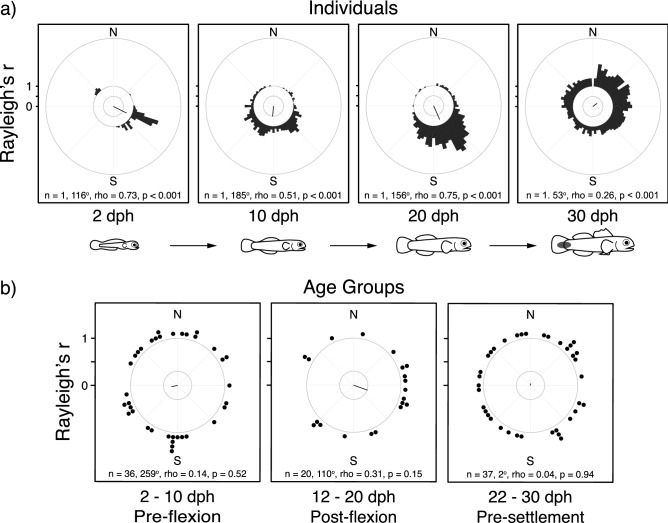
Ontogeny of orientation behavior towards a cardinal direction in lab-reared *E. lori* larvae deployed in situ in the DISC. (**a**) Randomly selected orientation plots for an individual larva at ages 2, 10, 20, and 30 days post-hatch (dph): grey bars indicate the frequency that a larva’s position was recorded within a 5° bin, and lines from the origin indicate the larva’s mean bearing. (**b**) Orientation plots comparing the mean bearing among larvae binned in three equal age groups: dots indicate the mean bearings of individual larvae, and lines from the origin indicate the mean bearing and rho-value among larvae in each age group.

To determine if lab-reared *E. lori* larvae demonstrate the ability to orient their movements, a Rayleigh test of uniformity was used to determine whether the subsampled positions of individual larvae deployed in the DISC were non-randomly distributed (see methods for details). The mean bearing and variance around the mean bearing (i.e., rho-value) were calculated (“Circular” package in R)^65,66^ for larvae that demonstrated positions that were non-randomly distributed. Data showed that at all ages tested (2–30 days post-hatch; dph, Fig. 4a), most larvae swam directionally (i.e., they oriented). The proportion of larvae that swam directionally was high (77.5% of larvae; 93 out of 120) and this did not change with age (Χ^2^ = 0.95, df = 1, *p* = 0.33). This result is consistent with previous studies that evaluated the orientation behavior of young larvae followed by divers^6,67^. These in situ experimental results indicate that pre-flexion *E. lori* larvae are able to orient their swimming within 2 days of hatching (i.e., the youngest age at which their behavior could be feasibly tested).

To determine whether larvae orient toward a common bearing, the mean bearings of all orienting larvae were pooled in a second order Rayleigh test. A common mean cardinal bearing was not present among larvae (i.e., the pooled mean bearings of all larvae in a cardinal frame of reference were not significantly different from a random distribution; all larvae: 2–30 dph, n = 93 larvae, *p* = 0.96, Table 1). In addition, a common mean cardinal bearing was not found among larvae when they were divided into three meaningful age groups (pre-flexion: 2–10 dph, n = 36 larvae, *p* = 0.52; post-flexion: 12–20 dph, n = 20 larvae, *p* = 0.15; and pre-settlement: 22–30 dph, n = 37 larvae, *p* = 0.94; Fig. 4b; Table 1)^68^. Larvae in each age group also did not orient with respect to a location-dependent cue, such as the direction of their natal reef (defined as the mid-point of the sponge transect on which the larvae were collected), or with respect to location-independent cues, such as current direction or wind direction (Table 1). One exception was that pre-settlement larvae oriented toward the sun azimuth (Table 1). While in situ sun compass orientation has been documented in other fish species (e.g., Ref.^17,63^), this study provides the first observation that sun compass orientation behavior develops late in the larval phase of a coral reef fish. At our study site, north of the equator, this behavior would result in pre-settlement larvae swimming in a general southerly direction, but the exact bearing would vary as the sun changes position throughout the day.

**Table 1:** Summary of Rayleigh tests of uniformity evaluating hypotheses for the goal of larval orientation behavior among all larvae in the sample [0–30 days post-hatch (dph)], and among ontogenetically relevant age groups (2–10, 12–20, 22–30 dph).

Orientation hypothesis	Age group (dph, Mean ± SD)	Sample size (n)	Mean bearing (degrees, Mean ± SD)	*rho* value (*r*)	*p* value
**Cardinal direction**					
All ages	2–30	93	145.2 ± 2.8	0.02	0.96
Age groups	2–10	36	258.8 ± 2.0	0.14	0.52
	12–20	20	109.7 ± 1.5	0.31	0.15
	22–30	37	2.1 ± 2.5	0.04	0.94
**Current direction**					
All ages	2–30	84	204.5 ± 2.17	0.10	0.47
Age groups	2–10	32	193.8 ± 1.95	0.15	0.50
	12–20	19	288.7 ± 2.63	0.03	0.98
	22–30	33	209.7 ± 2.15	0.10	0.72
**Wind direction**					
All ages	2–30	93	122.1 ± 2.39	0.06	0.74
Age groups	2–10	36	167.5 ± 1.87	0.17	0.34
	12–20	20	34.1 ± 1.65	0.25	0.28
	22–30	37	159.8 ± 2.69	0.03	0.97
**Sun azimuth**					
All ages	2–30	93	100.6 ± 2.07	0.12	0.28
Age groups	2–10	36	184.9 ± 1.81	0.20	0.25
	12–20	20	1.5 ± 1.95	0.15	0.65
	22–30	37	79.6 ± 1.53	0.31	0.03*
**Natal reef (transect)**					
All ages	2–30	93	8.9 ± 2.31	0.07	0.64
Age groups	2–10	36	7.0 ± 1.77	0.21	0.20
	12–20	20	141.7 ± 1.77	0.21	0.41
	22–30	37	309.8 ± 2.19	0.09	0.75

*Indicates a significant *p* value.

The ontogenetic transition that a pelagic larva goes through, from exhibiting a passive mode of dispersal to employing an active mode of dispersal, requires the development of both sufficient swimming abilities and the presence of sensory organs that can effectively detect environmental cues to initiate orientation behaviors in the open ocean^15,24,69^. Our study has revealed that the structural, and presumably, functional attributes of the developing sensory organs of *E. lori* were sufficient to provide sensory inputs that resulted in directional swimming behavior in larvae as early as 2 dph (Fig. 1). Further, the proportion of 2 dph larvae that swam directionally was quite high (77.5%), and this did not change throughout subsequent larval development. These results provide compelling evidence that *E. lori* larvae are equipped with sensory systems and directional swimming abilities that should allow them to actively influence their dispersal throughout their entire larval phase, which may help to explain the restricted pattern of dispersal for this species^36^.

### Implications for larval dispersal

Generally, location-independent cues, such as earth’s magnetic field^70^ or sun position^17,18,63^, are thought to provide information that could allow larvae to orient. In contrast, location-dependent cues, such as acoustic stimuli propagating from a reef, are considered to provide information that could allow larvae to move toward a geographic location. Indeed, many studies have demonstrated that late-stage fish larvae can use both location-dependent and location-independent cues to guide their movements^14–18,20,71,72^. While we did not explicitly test which sense or senses *E. lori* larvae use to orient their swimming behavior, our data indicate that pre-settlement, lab-reared larvae use a sun compass to orient. It is likely that *E. lori* larvae use multimodal sensory integration (processing of inputs from multiple sensory modalities simultaneously or sequentially through the larval phase) resulting in orientation behavior.

**Figure 5:**
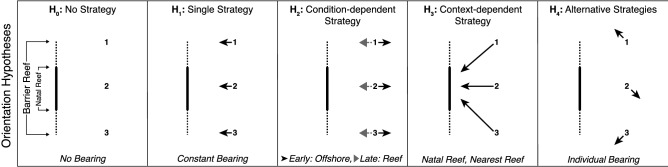
Alternative hypotheses for the goal of larval orientation behavior. (H_0_) No strategy, individual larvae may not swim directionally. (H_1_) Single strategy, all larvae share a common, constant bearing. (H_2_) Condition-dependent strategy, the optimal bearing changes depending on the ontogenetic or physiological state of individual larvae. (H_3_) Context-dependent strategy, the optimal bearing depends on an individual’s location. (H_4_) Alternative strategies, the optimal bearing depends on the frequency with which that bearing is adopted by other individuals in the population. Numbers—indicate the position of three hypothetical larvae with respect to a linear reef. Arrows—indicate the expected direction of orientation for each larva.

Once larvae are able to detect and interpret sensory cues, what orientation strategies do they use to reach a suitable settlement habitat? Larvae will have the greatest influence on their dispersal trajectory if they swim directionally^73^ starting early during the larval phase^22^. Thus, one needs to consider what larvae are swimming toward. Directional swimming behavior is expected to enhance survival by reducing the probability that larvae are advected away from potential settlement habitat^21,22^. To achieve this, larvae could adopt one or more orientation strategies that vary in complexity (Fig. 5). The simplest scenario is a single strategy, in which all larvae have the ability to swim along a common, constant bearing (i.e., orientation; Fig. 5: H_1_). For example, if *E. lori* larvae develop in the water column offshore from the Belizean Barrier Reef and swim to the west, they would move toward reef habitat regardless of their starting position and could then seek out their preferred sponge hosts^37^. Alternatively, a more complex condition-dependent orientation strategy may be used where the optimal bearing of a larva changes depending on its overall ontogenetic stage, the structure and function of one or more sensory systems, or its physiological state (Fig. 5: H_2_). For example, swimming to the east early in the larval phase may allow larvae to encounter higher densities of planktonic prey and escape the threat of planktivorous reef-based predators. However, later during the larval phase, swimming to the west may increase the probability of locating appropriate settlement habitat.

A natal reef represents high quality settlement habitat that has resources suitable for the survival and reproduction of post-settlement fishes. Assuming that the habitat is not saturated and the chance of inbreeding is low, larvae that adopt a context-dependent strategy and swim toward their natal reef may increase their potential for reaching settlement habitat that can ultimately maximize their fitness (e.g., true navigation toward their natal reef; Fig. 5: H_3_). Another context-dependent strategy would be for larvae to swim toward the nearest reef, which may increase the potential for reaching suitable settlement habitat if a larva had been advected away from its natal reef (e.g., true navigation toward the nearest reef).

Finally, larvae may employ alternative or mixed orientation strategies that vary among individuals within a population. From the parents’ perspective, offspring that employ alternative orientation strategies may maximize their opportunity to colonize available settlement habitat^74^. The ultimate effect that the different orientation strategies have on fitness will depend on the frequency with which each is used by individuals in the population (Fig. 5: H_4_)^75,76^. If their mean bearing remains constant throughout the larval phase, a portion of individuals would swim substantial distances toward nearby reef habitat, thus increasing their chances of detecting reef-based cues that could further facilitate successful settlement^77^, and another portion of individuals would move toward distant reefs, increasing their chances of exploiting new habitats. If these different orientation strategies are heritable, then the proportion of individuals that adopt “retentive” verses “dispersive” bearings would influence their relative fitness, and in turn, the frequency of each strategy that is used in the population^74,78^.

To determine what reef fish larvae are swimming toward, these alternative hypotheses will need to be tested by deploying larvae at different locations relative to their potential settlement habitat (e.g., the natal reef, or nearest reef) and in different environmental and ecological contexts (e.g., depths, distances from the reef, times of day, tidal phases). Here, we used wild-caught larvae, collected as they hatched from their natal tube sponge and subsequently reared in a field-based lab located on South Water Caye, Belize. While the results must be interpreted with this in mind, we found that most lab-reared *E. lori* larvae swam directionally (Fig. 4a) but that the mean bearing revealed by their orientation behavior varied among individuals (Fig. 4b). The lack of a common mean bearing among lab-reared individuals suggests either that the larvae tested may have been employing alternative orientation strategies (Fig. 5: H_4_), or that the rearing environment provided in the lab influenced their ability to orient along a common mean bearing. Critically testing and discriminating among these hypotheses will require further work.

## Conclusion

Over the past decade, biophysical models have become essential tools for the design of marine reserve networks that seek to optimize connectivity in order to enhance coral reef resilience^80–83^. The integration of larval orientation behavior into biophysical models is necessary to accurately predict larval dispersal trajectories and the resultant patterns of dispersal and population connectivity^42,84,85^. The current study has shown that larvae are equipped with sensory systems, swimming abilities and orientation behaviors that could allow them to influence their dispersal beginning shortly after hatch and continuing throughout the larval phase. There continues to be a pressing need to better understand the role of larval behavior in shaping patterns of dispersal and population connectivity, not just in reef fishes, but in a wide range of marine animal species.

## Materials and methods

### Larval rearing for morphological analyses and DISC trials

*Elacatinus lori* embryos were collected using a slurpgun from the internal wall of 135 *Aplysina fistularis* located along a 650 m long transect, offshore from South Water Caye, Belize during May–August 2016. The embryos hatched immediately upon collection and the newly-hatched larvae were transferred in water collected in and around host sponges to a flow-through seawater lab at the International Zoological Expeditions (IZE) field station for rearing. Larvae collected from multiple sponges on the same day were acclimated to a 76 L cylindrical black rearing bin, filled with ~ 50% water collected with larvae and ~ 50% filtered seawater. Larvae were fed once daily with natural prey items (wild-caught plankton [55–150 µm]) and HUFA enriched-rotifers (*Brachionus rotundiformis*, 15 mL^−1^) following established methods^35^. Beginning 3 days post-hatch (dph), water was exchanged (at a rate of 250 mL min^−1^ for 2 h each morning) using seawater pumped from the reef lagoon and detritus was siphoned from the rearing bins. The lab was provided with a 14 L : 10 D light cycle, and water quality in the rearing bins was maintained at a salinity of 33–35 ppt, temperature of 27–28 °C, pH of 8.0–8.3, NH_3_ concentrations of 0–0.25 ppm, NO_2_ concentrations of 0 ppm, and NO_3_ concentrations of 0 ppm.

To provide additional specimens for morphological analyses, larvae were also obtained from wild-caught, captive breeding pairs of *E. lori* maintained in a recirculating seawater system at Boston University. Spawning shelters were checked each morning for clutches of eggs. On the night prior to hatching an individual clutch was transferred to a black, cylindrical 76-L rearing bin and a gentle stream of air was directed over the eggs to stimulate hatching. Upon hatching, larvae were fed *HUFA*-enriched rotifers *B. rotundiformis* (15 mL^−1^), decapsulated *Artemia* nauplii (3 mL^−1^) and the water was tinted with *Nannochloropsis* algal paste (Rotigreen Nanno, Reed Mariculture, USA). All methods were approved by, and performed in accordance with the guidelines and regulations of, the Belize Fisheries Department and the Boston University IACUC (IACUC protocols: 13–021 and 10–036) and carried out in compliance with ARRIVE guidelines.

### Morphological methods

Development of the visual, auditory, olfactory and gustatory systems were assessed using histological analyses. *Elacatinus lori* larvae reared in the field-based lab in Belize in 2015 and 2016 (n = 33, 0–44 dph, 3 mm notochord length [NL]—11 mm standard length [SL]) and wild-caught post-settlement juveniles collected in Belize in 2015 and 2016 (n = 5, 9.5–14 mm SL) were fixed in 10% formalin in seawater, prepared for glycol methacrylate resin histology (serial sections; 5 µm thickness) and stained with cresyl violet^26,27^. Larvae reared at Boston University in 2015 (n = 4, 0–30 dph, 3 mm NL–7 mm SL) and post-settlement juveniles wild-caught in Belize in 2011 (n = 8, 9–17 mm SL) were fixed in 10% formalin in seawater and prepared for paraffin histology (serial sections; 8 µm thickness) and stained following the HBQ protocol (highlighting cell nuclei, bone and cartilage^26,27^).

The number and distribution of neuromasts were visualized using a vital fluorescent mitochondrial stain (0.0024% [4-di-2-ASP (4-(4-(diethylamino) styryl)-N-methylpyridinium iodide] in seawater^27^) in a total of 22 live, anaesthetized (0.02% buffered MS-222) *E. lori* larvae (0–38 dph) reared at Boston University (0 dph [n = 4], 10 dph [n = 4], 20 dph [n = 5], 31 dph [n = 5], and 38 dph [n = 5]; 3 mm NL to 9.5 mm SL) and wild-caught adults (n = 4, 42–62 mm SL). Neuromasts in live fish were imaged on a Nikon SMZ 1500 dissecting microscope equipped with a GFP filter set.

The number and structure of neuromasts and taste buds were further visualized using scanning electron microscopy. *Elacatinus lori* lab-reared at Boston University in 2015 (n = 10, 0–45 dph, 2.5 mm NL-11 mm SL), *E. lori* lab-reared in Belize in 2016 (n = 24, 0–44 dph, 3–8 mm SL), and wild-caught post-settlement juveniles collected in Belize in 2011 (n = 5, 9–18 mm SL) were fixed in 4% formalin in seawater and prepared for scanning electron microscopy (critical point dried out of liquid CO_2_ and sputter coated with palladium) and viewed with a Zeiss NTS Supra 40VP SEM at 3 kV and a working distance of ~ 10 mm^26,27^.

### DISC methods

To investigate the ontogeny of swimming and orientation behavior in situ, larvae reared in the field-based lab in Belize were deployed in a drifting in situ chamber (DISC^12^, Fig. 2c) composed of a symmetrical acrylic frame that supports a cylindrical arena (20 cm diameter, 10 cm height). The bottom of the arena was made of clear acrylic, the top was made of translucent, 400 µm mesh, open to the ambient water and environmental cues. To prevent the delicate larvae from being damaged on the mesh during deployment to depth, the side walls of the arena were made of opaque black foam material. Sensors attached to the frame of the DISC recorded the salinity, temperature, depth (DST-CTD, Star-Oddi, Iceland), light level (HOBO pendant, Onset Computer Corporation, USA), and rotation (3 analog compasses and 1 custom digital compass) of the DISC throughout each deployment. A GPS equipped float suspended the DISC at a constant depth while recording its location, and a drogue attached to the bottom of the DISC coupled the apparatus with the subsurface current. A small boat was used to simultaneously deploy two DISCs ≤ 1 km offshore from the larvae’s natal reef (i.e., the transect where the larvae were collected) during ebb tides (Fig. 2b). At this distance, the far field (i.e., pressure) components of sound propagating from the reef are expected to be available to larvae deployed in the DISC^86^. A previous study found that larval orientation behaviors were disrupted when the sun was near its zenith (1200–1400)^10^. Therefore, most deployments were conducted between 0700 and 1200 or 1400 and 1800 h.

*Elacatinus lori* larvae reared in the field-based lab in Belize were tested in the DISC every 2 days throughout development, from 2 to 30 days post hatch, for a total of 120 successful deployments (Fig. 2a, SI Table 2). To begin each day of trials, larvae were sampled haphazardly from the rearing bin, loaded individually into opaque perforated vials, and transported to the deployment site in a cooler. To deploy larvae into the DISC, SCUBA divers descended slowly with the vials to 10 m (below the surface turbulence) then gently released an individual larva into the DISC’s arena. The divers ensured that the larva was exhibiting normal swimming behavior before lowering the DISC slowly to its final deployment depth (19 m, Fig. 2b). If abnormal swimming behavior was observed (e.g., laying on the bottom, loss of balance, whirling), the larva was removed from the arena and replaced with a different individual. To avoid producing auditory, visual, or chemosensory cues that could bias the trials, the divers exited the water after deploying a larva in each DISC, the boat was relocated offshore (~ 500 m), and the motors were shutoff. Each trial lasted 20 min: 5 min of acclimation and 15 min of data acquisition. During a trial, larval behavior was recorded continuously using a GoPro HERO4 mounted below the arena (GoPro, USA).

### Statistical analysis

Larval orientation behavior was determined by analyzing the video recordings of each trial. From these videos, the position of a larva within the DISC was subsampled from individual frames at 1 s intervals using the “discr” package in R^66^. If the positions were found to be non-randomly distributed using a Rayleigh test of uniformity, then the larva’s mean orientation bearing and rho-value (i.e., a measure of variance around the mean bearing) were calculated (93 of 120 deployments; SI Table 2). Deployments in which the larva’s positions within the DISC were not significantly different from a random distribution were eliminated from the dataset (5 of 120 deployments; SI Table 2). The DISC is designed to rotate slowly as it drifts with the current. Therefore, in addition to analyzing the videos in the camera’s frame of reference, the video data was corrected by the synchronized compass readings and analyzed in the cardinal frame of reference. Comparing analyses in the camera’s frame of reference and cardinal frame of reference allowed for discrimination of artifactual behavior, where larvae were attracted to a certain part of the arena as the DISC rotated, from genuine orientation behavior, where larvae oriented with respect to a cardinal direction regardless of the DISC’s rotation^12^. Larvae with a higher rho-value (i.e., lower variance around the mean bearing) when analyzed in the camera’s frame of reference compared to the cardinal frame of reference were identified as having exhibited a behavioral bias toward a component of the DISC and were eliminated from the dataset (22 of 120 larvae; SI Table 2)^61^. In addition to the mean bearing and rho-value, each individual larva’s mean instantaneous swimming speed, and mean turning angle (i.e., the angle produced by three successive points on a larva’s trajectory) were calculated.

To test the hypothesis that swimming and orientation behaviors change throughout the larval phase, the relationship of larval age with instantaneous swimming speed and mean turning angle were evaluated using linear regression. Data from larvae reared prior to this study in the same field-based lab revealed that there was a strong linear relationship between size and age in this species (SI Fig. 1, SI Table 3). Based on this analysis and methodological constraints associated with recollecting and measuring larvae that were deployed in situ, we chose to use age, rather than size, as a proxy for development when analyzing data collected using the DISC. The proportion of larvae that oriented significantly along a mean bearing was compared among ages using a logistic regression. To test the hypothesis that larvae share a common mean bearing at the population level, the data were evaluated using a second order Rayleigh test among all larvae tested (2–30 dph), among larvae at each age tested (2, 4, 6, … , 26, 28, 30 dph), and among three ontogenetically relevant age groups (pre-flexion: 2–10, post-flexion: 12–20, and pre-settlement: 22–30 dph). Finally, to test the hypothesis that larvae orient with respect to cues from their environment, we evaluated whether the population orients with respect to sun azimuth, current direction, wind direction, or their natal reef.

## Supplementary Information

Supplementary Information.

## Data Availability

All datasets used in the analyses presented in this study are available from the Biological & Chemical Oceanography Data Management Office (BCO-DMO) website (https://www.bco-dmo.org/project/651265). The open-source R-software package “discr”, which was used to process the data from DISC deployments, is available from the GitHub repository (https://github.com/jiho/discr)^61^.
