# Supplementary material for: An integrative investigation of sensory organ development and orientation behavior throughout the larval phase of a coral reef fish
Source: Sci Rep. 2021 Jun 11;11:12377. doi: 10.1038/s41598-021-91640-2 (PMC8196062; doi:10.1038/s41598-021-91640-2)
Supplement: Supplementary file 1 — Supplementary Information. [file 41598_2021_91640_MOESM1_ESM.docx]

**An integrative investigation of sensory organ development and orientation behavior throughout the larval phase of a coral reef fish**

John E. Majoris^1,2,*^; Matthew A. Foretich^3^; Yinan Hu^4,5^; Katie R. Nickles^4^, Camilla L. Di Persia^3^; Romain Chaput^3^; E. Schlatter^1,6^; Jacqueline F. Webb^4^; Claire B. Paris^3^; Peter M. Buston^1^

*^1^ Department of Biology and Marine Program, Boston University, Boston MA 02215 USA*

*^2^ Current Address: Division of Biological Sciences and Engineering, Red Sea Research Center, King Abdullah University of Science and Technology, Thuwal 23955-6900, Kingdom of Saudi Arabia*

*^3^ Department of Ocean Sciences, University of Miami’s Rosenstiel School for Marine and Atmospheric Science, Miami FL 33149 USA*

*^4^ Department of Biological Sciences, University of Rhode Island, Kingston RI 02881 USA*

*^5^Current Address: Department of Biology, Boston College, Chestnut Hill MA 02467 USA*

*^6^ Department of Biology, Colorado State University, Fort Collins CO 80523 USA*

*^*^ Corresponding Author: j.e.majoris@gmail.com****;*** *(+966) 0544700263*

**SI Table 1.** Metadata for the photos of specimens included in Figure 1. NL *=* notochord length (mm); SL = standard length (mm). Unk. = unknown.

| **Panel ID** | **Sensory System** | **Stage** | **Age** | **Size** | **Metric** | **Panel Scale Bar** | **Location** | **Sid** |
| --- | --- | --- | --- | --- | --- | --- | --- | --- |
| A1 | Live Larva | Hatch | 0 | 3.5 | NL | 500 $\mu m$ | Lab-raised | Right side, image transposed |
| B1 | Lateral Line | Hatch | 0 | 3 | NL | NA | Lab-raised | Right side, image transposed |
| C1 | Visual | Hatch | 0 | 3 | NL | 50 $\mu m$ | Lab-raised | Right eye, image transposed |
| D1 | Auditory | Hatch | 0 | 3 | NL | 50 $\mu m$ | Lab-raised | Left side |
| E1 | Olfactory | Hatch | 0 | 3 | NL | 50 $\mu m$ | Lab-raised | Left side |
| F1 | Gustatory | Hatch | 0 | 3 | NL | NA | Lab-raised | NA |
| A2 | Live Larva | Flexion | 10 | 5.0 | SL | 500 $\mu m$ | Lab-raised | Right side, image transposed |
| B2 | Lateral Line | Flexion | 10 | 4.5 | SL | NA | Lab-raised | Right side, image transposed |
| C2 | Visual | Flexion | 10 | 5 | SL | 50 $\mu m$ | Lab-raised | Right eye, image transposed |
| D2 | Auditory | Flexion | 10 | 4.5 | SL | 50 $\mu m$ | Lab-raised | Left side |
| E2 | Olfactory | Flexion | 12 | 4.5 | SL | 50 $\mu m$ | Lab-raised | Left side |
| F2 | Gustatory | Flexion | 10 | 4.5 | SL | NA | Lab-raised | NA |
| A3 | Live Larva | Post-flexion | 20 | 7.5 | SL | 500 $\mu m$ | Lab-raised | Right side, image transposed |
| B3 | Lateral Line | Post-flexion | 20 | 6 | SL | NA | Lab-raised | Left side |
| C3 | Visual | Post-flexion | 20 | 5 | SL | 50$\mu m$ | Lab-raised | Right eye, image transposed |
| D3 | Auditory | Post-flexion | 20 | 6 | SL | 50 $\mu m$ | Lab-raised | Left side |
| E3 | Olfactory | Post-flexion | 20 | 5.5 | SL | 50 $\mu m$ | Lab-raised | Left side |
| F3 | Gustatory | Post-flexion | 20 | 6.5 | SL | NA | Lab-raised | NA |
| A4 | Live Larva | Pre-settlement | 28 | 9 | SL | 500 $\mu m$ | Lab-raised | Right side, image transposed |
| B4 | Lateral Line | Pre-settlement | 31 | 9 | SL | NA | Lab-raised | Left side |
| C4 | Visual | Pre-settlement | 30 | 8 | SL | 50 $\mu m$ | Lab-raised | Right eye, image transposed |
| D4 | Auditory | Pre-settlement | 28 | 7 | SL | 50 $\mu m$ | Lab-raised | Left side |
| E4 | Olfactory | Pre-settlement | 28 | 7 | SL | 50 $\mu m$ | Lab-raised | Left side |
| F4 | Gustatory | Pre-settlement | 30 | 7 | SL | NA | Lab-raised | NA |
| A5 | Live Larva | Post-settlement | 38 | 9.5 | SL | 500 $\mu m$ | Lab-raised | Right side, image transposed |
| B5 | Lateral Line | Post-settlement | 38 | 9 | SL | NA | Lab-raised | Left side |
| C5 | Visual | Post-settlement | Unk. | 14 | SL | 50 $\mu m$ | Wild-caught | Right eye, image transposed |
| D5 | Auditory | Post-settlement | 34 | 9 | SL | 50 $\mu m$ | Lab-raised | Left side |
| E5 | Olfactory | Post-settlement | 44 | 11 | SL | 50 $\mu m$ | Lab-raised | Left side |
| F5 | Gustatory | Post-settlement | Unk. | 15 | SL | NA | Wild-caught | NA |

**SI Table 2.** Number of larvae deployed in the DISC at each age that oriented significantly, did not orient, or artifacts (i.e., larvae that continually oriented toward a certain part of the arena as the DISC rotated).

| Age (dph) | Significant Orientation (n) | Non-significant  Orientation (n) | Artifacts (n) |
| --- | --- | --- | --- |
| 2 | 8 | 0 | 2 |
| 4 | 4 | 2 | 0 |
| 6 | 9 | 0 | 2 |
| 8 | 9 | 0 | 1 |
| 10 | 6 | 0 | 2 |
| 12 | 4 | 0 | 0 |
| 14 | 1 | 0 | 0 |
| 16 | 5 | 0 | 1 |
| 18 | 3 | 0 | 1 |
| 19 | 1 | 0 | 0 |
| 20 | 6 | 1 | 2 |
| 22 | 5 | 0 | 1 |
| 24 | 4 | 0 | 3 |
| 26 | 13 | 0 | 0 |
| 28 | 7 | 1 | 4 |
| 30 | 8 | 1 | 3 |
| Total | 93 | 5 | 22 |


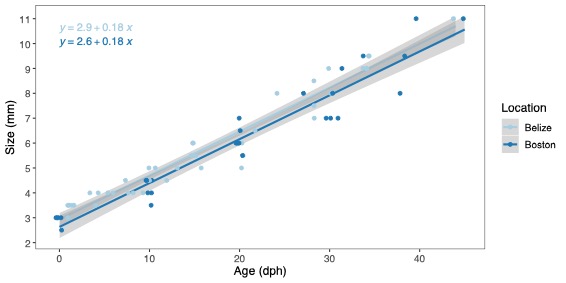


**SI Figure 1.** The relationship of age and size throughout development from hatching through settlement in lab-raised E. lori larvae. Measurements of notochord length or standard length were made to the nearest 0.5mm from lab-reared specimens that were preserved in 10% formalin in PBS and subsequently used for the morphological analyses presented in this manuscript, Hu et. al 2019 and Nickles et al. 2020. Specimens were obtained from clutches of larvae raised in Boston and Belize in 2015 and 2016. An ANCOVA analysis revealed that there was no significant different in the slope of the relationship of age and size among years or locations, but that on average larvae raised in Boston were 0.3mm smaller at any given age than larvae raised in Belize. Data points are jittered to reduce overplotting. Blue lines represent the fit of the ANCOVA model, and grey shading represents the 95% confidence intervals.

**SI Table 3.** Summary of the results of an analysis of covariance (ANCOVA) that investigated the relationship of the size of lab-raised larvae with multiple independent variables. Significant *P-*values are highlighted in bold.

| **Variable** | **df** | **SS** | **MS** | ***F*** | ***P-value*** |
| --- | --- | --- | --- | --- | --- |
| **Age** | 1 | 346.96 | 346.96 | 1121.73 | **<0.001** |
| **Location** | 1 | 1.56 | 1.56 | 5.05 | **0.027** |
| **Year** | 1 | 0.74 | 0.74 | 2.39 | 0.127 |
| **Location * Year** | 1 | 0.3 | 0.3 | 0.98 | 0.325 |
| **Residuals** | 66 | 20.41 | 0.31 |  |  |
